# Death of Dr. Cochran

**Published:** 1853-10

**Authors:** 


### DEATH OF DR. COCHRAN.

Dr. WILLIAM COCHRAN, one of the oldest physicians of this city, died of yellow fever, at Biloxi, on the 20th of August. The deceased was on a visit to his son, Dr. WILLIAM W. COCHRAN, when he was seized with the fatal epidemic. He has been a practitioner in Kentucky for many years, and was greatly esteemed for his excellent social and professional qualities. His manners were those of an old Virginia gentleman, urbane, dignified, and courteous, and his impulses warm, generous and noble. For several years past he had retired very much from the active duties of his profession, but he leaves behind him two sons, worthy representatives in the calling which he adorned. We hope, in a future number, to be able to give a sketch of the life and professional services of Dr. COCHRAN.

The following proceedings on the part of the physicians of Louisville took place when the intelligence of his death reached the city:

"At a meeting of the physicians of Louisville, held on the 5th ult., on motion of Dr. KNIGHT, Dr. L. P. YANDELL was called to the chair, and Dr. B. I. RAPHAEL appointed Secretary.

"The object of the meeting having been stated by the CHAIRMAN, it was moved that a Committee be appointed to draft resolutions in conformity therewith.

"The following gentlemen were appointed: Drs. B. I. RAPHAEL, MANDEVILLE THUM, and WM. H. MILLER, who, after retiring a short time, presented the following resolutions, which were unanimously adopted:

"WHEREAS, It having pleased Almightly God to remove from our midst our venerable friend and fellow-practitioner, Dr. WM. COCHRAN, who departed this life after a very brief illness, at Biloxi, Miss., on the 20th August; and in view of the many virtues which endeared him to all who knew him, therefore,

*Resolved*, That we have received with profound regret the intelligence of the death of Dr. COCHRAN.

*Resolved*, That in the death of Dr. COCHRAN the medical paofession has sustained the loss of a venerable member, and a firm and undeviating friend.

*Resolved*, That we deeply sympathize with his bereaved family in the irreparable loss which they have sustained by this dispensation of Divine

Providence, and that the Secretary be directed to transmit them a copy of these proceedings.

"*Resolved*, That these proceedings be published in the daily papers of the city, in the *Western Journal of Medicine and Surgery*, and the *Transylvania Journal of Medicine*.

"On motion, the meeting adjourned.

"L. P. YANDELL, Chairman.

"B. I. Raphael, Secretary."

